# The complete genome sequence and analysis of vB_VorS-PVo5, a *Vibrio* phage infectious to the pathogenic bacterium *Vibrio ordalii* ATCC-33509

**DOI:** 10.1186/s40793-016-0166-6

**Published:** 2016-07-04

**Authors:** Alex Echeverría-Vega, Pablo Morales-Vicencio, Camila Saez-Saavedra, Janja Ceh, Rubén Araya

**Affiliations:** Programa de Doctorado en Ciencias Aplicadas, Mención Sistemas Marinos Costeros, Facultad de Ciencias del Mar y Recursos Marinos, Universidad de Antofagasta, Angamos 601, Antofagasta, Chile; Laboratorio de Microbiología Costera, Facultad de Ciencias del Mar y Recursos Biológicos, Universidad de Antofagasta, Angamos 601, Antofagasta, Chile; Instituto de Ciencias Naturales Alexander von Humboldt, Facultad de Ciencias del Mar y Recursos Biológicos, Universidad de Antofagasta, Angamos 601, Antofagasta, Chile

**Keywords:** Short genome report, Bacteriophage, *Vibrio ordalii*, Siphoviridae, DS-DNA virus

## Abstract

The bacterium *Vibrio ordalii* is best known as the causative agent of vibriosis outbreaks in fish and thus recognized for generating serious production losses in aquaculture systems. Here we report for the first time on the isolation and the genome sequencing of phage vB_VorS-PVo5, infectious to *Vibrio ordalii* ATCC 33509. The features as well as the complete genome sequence and annotation of the *Vibrio* phage are described; vB_VorS-PVo5 consists of a lineal double stranded DNA totaling ~ 80.6 Kb in length. Considering its ability to lyse *Vibrio ordalii* ATCC 33509, the phage is likely to gain importance in future aquaculture applications by controlling the pathogen and as such replacing antibiotics as the treatment of choice.

## Introduction

The Chilean coast is characterized by the Humboldt Current System, a cold, low-salinity ocean current that is considered one of the most productive marine ecosystems on Earth. Cold, nutrient rich waters are constantly upwelled into the photic zone providing sustenance for primary producers [1, 2]. This environment offers highly favorable natural conditions for the growth of heterotrophic bacteria [3], e.g., for *Vibrio* species, known as the main pathogenic bacterial group in Chilean aquaculture [4, 5]. One of the most frequently isolated marine *Vibrio* species in the salmon industry is *Vibrio ordalii* which has been described as highly pathogenic for larvae reared in hatcheries [6–9], and therefore significantly contributes to production losses. In order to prevent diseases and control infections in fish farms, the intensive use of a wide variety of antimicrobials is applied. However, the poor management of such treatments, e.g., the use of antibiotics in discrete doses as a prophylactic therapy [10, 11], has caused enormous damage to the environment [12–14]. Moreover, the increasing development of antimicrobial resistance in natural bacterial communities [10, 15, 16], calls for stricter regulations of antibiotic use [17]. As a consequence, the interest in phage therapy as an alternative control for bacteria in aquaculture systems has recently re-gained momentum [18–21].

In our quest to find a natural control for *V. ordalii* in aquaculture, we focused our research on isolating phages potentially effective against the pathogen, surveying various marine sources, e.g., sea water, sediment and intertidal filter organisms. Amongst others we tested the filter-feeding *Perumytilus purpuratus* (Lamarck, 1819), an intertidal mussel common to the northern Chilean coast and a promising source organism for phages as it uptakes and concentrates local microbiota in its gut system. We succeeded in isolating and identifying vB_VorS-PVo5 a novel marine phage belonging to the family Siphoviridae that causes lytic infections in the bacterium *V. ordalii*ATCC 33509 and therefore qualifies as a potent future candidate to control one of the most harmful bacteria in the aquaculture industry. The whole genome sequence of the phage was sequenced on an Illumina MiSeq platform and is described here, presenting the first report of an isolated and sequenced phage that infects the marine bacterium *Vibrio ordalii*.

## Organism information

### Classification and features

The *Vibrio* phage vB_VorS-PVo5 belongs to the Siphoviridae, a family of double-stranded DNA viruses in the order Caudovirales, and forms ~2-mm diameter plaques when infecting *V. ordalii* type strain ATCC 33509.

**Fig. 1:**
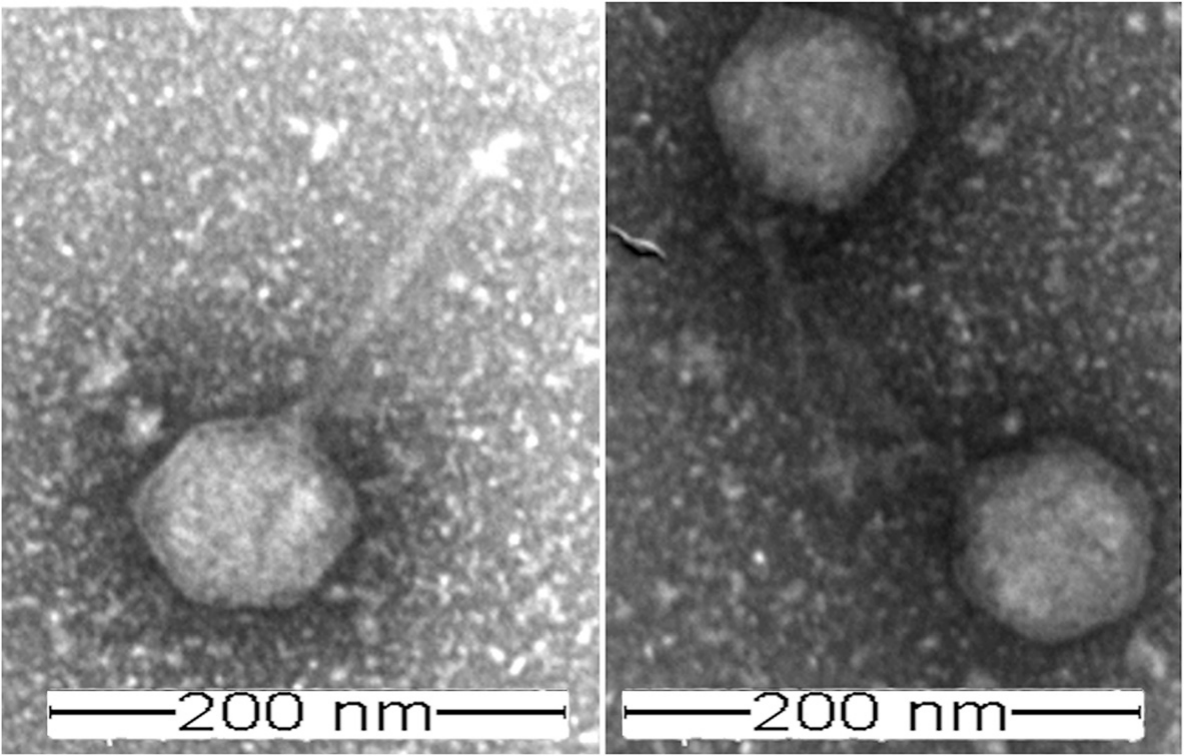
Transmission electron micrograph of *Vibrio* phage vB_VorS-PVo5. Scale bar = 200 nm

**Fig. 2:**
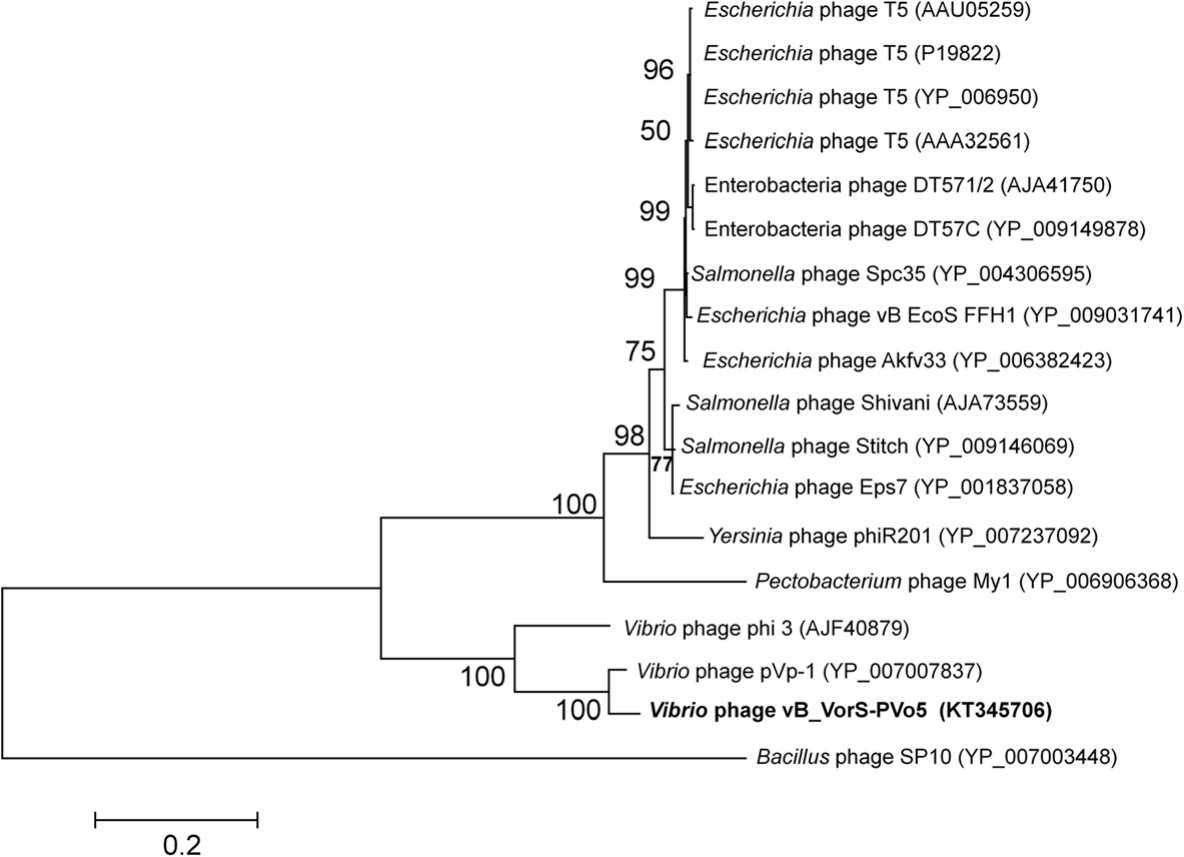
Phylogenetic tree highlighting the relatedness between the *Vibrio* phage vB_VorS-PVo5 (shown in bold) and other viruses. The tree is based on aligned sequences of DNA polymerases over 50 % similarity by BLASTP, using the *Bacillus* phage SP10 as the outgroup. The DNA polymerase sequence is 863 aa in length. The bootstrap consensus was set to 1000 replicates

The phage was isolated from macerated specimens of the mussel *Perumytilus purpuratus*, collected in the intertidal zone off the Antofagasta coast in Chile (23°42’00”S; 70°25’88”W). Transmission electron microscopy of purified phage particles (Fig. 1) revealed an icosahedral capsid (~85 nm diameter) and a distinguishable long tail (~150 nm length). The capsid encapsulates a linear double-stranded DNA genome of a length of 80,578 bp. An alignment of the DNA polymerase gene, a method commonly applied as a viral phylogenetic marker [22], demonstrated that vB_VorS-PVo5 phage clusters closely to (a) *Vibrio* phage pVp_1 GB JQ340389, a known predator of *V. parahaemolyticus*ATCC 33844 previously isolated from the Yellow Sea coast in Korea [23], and (b) *Vibrio* phage phi 3 GB AJF40879, associated with *Vibrio cholerae* 1051 and previously isolated in Russia (Fig. 2). All sequences were collected from NCBI and aligned using CLUSTALW [24]; the evolutionary analysis was inferred through the neighbor-joining method using MEGA6 [25] under auto settings.

## Genome sequencing information

### Genome project history

The bacteriophage vB_VorS-PVo5 was selected for genome sequencing based on its potential as a biological control agent for the pathogenic bacterium *Vibrio ordalii*; there are no previous reports describing the isolation and characterization of a lytic phage against this bacterium. The present study represents a first step towards better understanding the roles of bacteriophages in the ecology and virulence of *V. ordalii* and contributes to the limited data sets of vibriophage and podovirus genomes. All processes, including the isolation, multiplication, count and selection of phages, and their DNA extraction/purification were performed in the Laboratorio de Microbiología Costera at the University of Antofagasta (see following section for more details). The DNA sequencing and assembly was performed at the Molecular Research Laboratory MR-DNA (Shallowater, TX). The annotation and submission was performed in-house, using openly accessible informatics tools (see previous section for more details). The genome project was recorded in the NCBI database under the accession number PRJNA290661 and the genome annotation was deposited in GenBank under the accession number KT345706. A summary of the phage’s features is shown in Table 1 and detailed project information is given in Table 2.

**Table 1:** Classification and general features of *Vibrio* phage vB_VorS-PVo5

MIGS ID	Property	Term	Evidence code^a^
	Classification	Domain: viruses, dsDNA viruses, no RNA phage	TAS [39]
		Phylum: unassigned	
		Class: unassigned	
		Order: Caudovirales	TAS [39]
		Family: Siphoviridae	TAS [39]
		Genus: T5likevirus	TAS [39]
		Species: unassigned	
		(Type) strain: vB_VorS-PVo5 (KT345706*)*	
	Particle shape	Icosahedral head with a long tail	IDA
	Motility	non-motile	IDA
	Sporulation	Not reported	IDA
	Temperature range	14–28 °C	IDA
	Optimum temperature	25 °C	IDA
	pH range; Optimum	6.5–7.5	IDA
	Carbon source	Host cell	IDA
MIGS-6	Habitat	Oceanic, coastal	IDA
MIGS-6.3	Salinity	35 % NaCl (w/v)	IDA
MIGS-22	Oxygen requirement	Facultative aerobic	IDA
MIGS-15	Biotic relationship	Obligate intracellular parasite of *Vibrio ordalii*	IDA
MIGS-14	Pathogenicity	Lytic virus of *Vibrio ordalii*	IDA
MIGS-4	Geographic location	Chile/Antofagasta	IDA
MIGS-5	Sample collection	Jan 25 2014	IDA
MIGS-4.1	Latitude	23.65 S	IDA
MIGS-4.2	Longitude	70.5 E	IDA
MIGS-4.4	Altitude	0 m	IDA

^a^Evidence codes - IDA: Inferred from Direct Assay; TAS: Traceable Author Statement (i.e., a direct report exists in the literature); NAS: Non-traceable Author Statement (i.e., not directly observed for the living, isolated sample, but based on a generally accepted property for the species, or anecdotal evidence). These evidence codes were obtained from the Gene Ontology project [40]

**Table 2:** Project information

MIGS ID	Property	Term
MIGS 31	Finishing quality	Complete
MIGS-28	Libraries used	
MIGS 29	Sequencing platforms	Illumina Myseq
MIGS 31.2	Fold coverage	130
MIGS 30	Assemblers	NGEN (DNAstar)
MIGS 32	Gene calling method	PHAST server [29] Glimmer 2.1 [30]
	Locus Tag	AEO54
	Genbank ID	KT345706
	GenBank Date of Release	November 03 2015
	GOLD ID	Gp0120391
	BIOPROJECT	PRJNA290661
MIGS 13	Source Material Identifier	Personal culture collection
	Project relevance	Aquaculture

### Growth conditions and genomic DNA preparation

The phage multiplication was performed applying the double-layer agar plates method [26, 27]. *Vibrio ordalii* cells (conc. 5E + 06 cells/mL) in the soft layer of Tryptone Soy Agar, (Oxoid, UK) were subjected to six serial dilutions (1:10–1:1000000) of phage and the lysis plaques formed were counted to determine the total number of phage. For the DNA extraction each of three plates were inoculated with 1000 PFU phage and incubated for 16 h at 25 °C. Viral particles were re-suspended in 4 mL phage buffer [28] and incubated for 4 h with intervals of gentle shaking at 30 min. Subsequently the supernatant was transferred into 15 mL falcon tubes, a 1 mL chloroform solution was added, gently shaken for 30 s and centrifuged at 5000 rpm for 5 min. The product was filtered through 0.22 μm nitrocellulose filters (Merck-Millipore, Germany) to eliminate bigger cells. For the elimination of all external genomic content, DNase I (Thermo-Fisher, Germany) and RNase-A (Thermo-Fisher, Germany) were added at a final concentration of 5 units mL^−1^ each, for an incubation time of 30 min at 37 °C. Subsequently the flocculant PEG-80 was added at a 4:10 ratio and incubated overnight at 4 °C. Viral particles were pelleted in a centrifugation step at 10000 × g for 1 h at 4 °C. The supernatant was removed, the pellet dried for ~5 min under a sterile hood, a 50 μl of 10 units mL^−1^ Proteinase K (Thermo-Fisher, Germany) and phage buffer mix added, and incubated at 50 °C for 30 min to inactivate nucleases. The genomic DNA of the vB_VorS-PVo5 phage was extracted with a Phage DNA Isolation Kit (Norgen Biotek Corp., Canada), and evaluated and quantified with a UV–VIS spectrophotometer (BioTek Epoch, USA). In order to confirm the type of nucleic acid extracted, the product was digested separately in 1 unit/mL DNAse I and RNAse A, respectively. DNAse I, as opposed to RNAse A, degraded the extract, confirming the organism to be a DNA-containing phage.

### Genome sequencing and assembly

The genome was sequenced on an Illumina MiSeq platform at the MR-DNA Laboratory (Shallowater, TX). The library for each sample was prepared using a Nextera DNA Sample Preparation Kit (Illumina), following the manufacturer’s instructions. 2 × 130-bp paired-end reads allowed for an estimate of 50.000 output sequences of 287 bp length with 45,367 reads remaining after the quality filtering. The assemblage of quality-filtered reads was executed for the complete genome sequence, using the pipeline by MR-DNA and resulted in an average coverage of 130 fold. A single contig of 80,578 bp corresponding to the linear genome was assembled using NGEN (DNASTAR ®) by MR-DNA.

### Genome annotation

The prediction of open reading frames and the comparative analysis were performed combining two methods: the PHAST server [29] and Glimmer 2.1 [30]. For the assignment of protein functions to ORFs a combination of an automatic and a manual method was used, i.e., the PHAST server and BLASTp against the NCBI non-redundant database. Only homologues with E-values <1e-5 were present in the annotations. The tRNA genes were searched using the tRNAscan-SE 1.21 tool [31] and TMHMM [32], and SignalP [33] were used to predict transmembrane helices and signal peptides, respectively.

## Genome properties

**Fig. 3:**
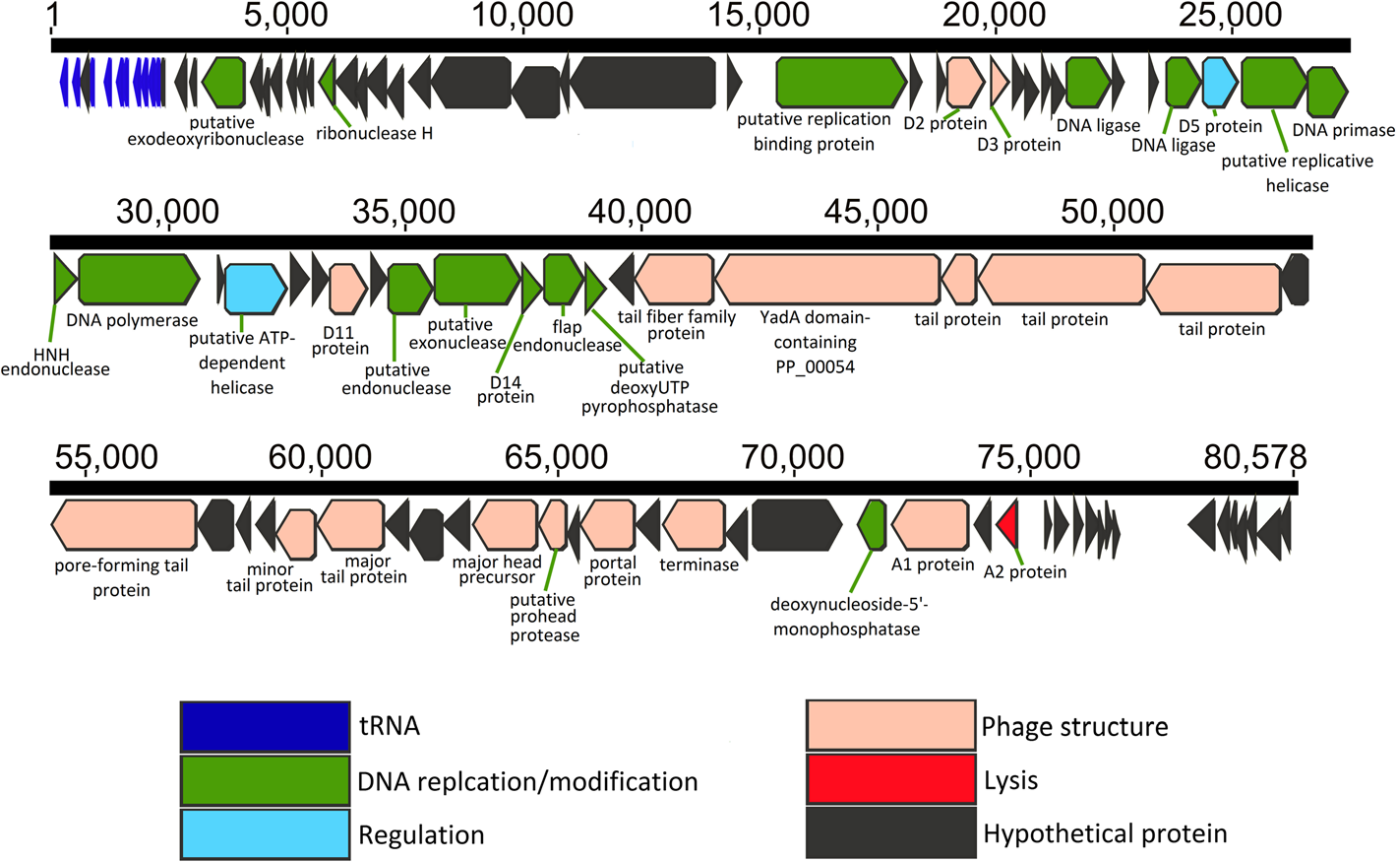
Gene map of the *Vibrio* phage vB_VorS-PVo5

The genome statistics are summarized in Table 3. The double-stranded and non-redundant DNA genome displayed a length of 80,578 bp with a G + C content of 40.55 %. A total of 10 tRNAs and 93 ORFs were identified, with no bacterial matches found. Putative functions of the identified ORFs were clustered by function [34], revealing DNA genes related to: metabolism (16), head/capsid proteins (7), phage tails (7), phage fibers and fiber assemblies (2), endolysin (1), and transcriptional regulation (1). The remaining 59 ORFs did not match any known function. The COG functional categories of identified genes are presented in Table 4, whereas the gene map is displayed in Fig. 3. The phylogenetic tree was constructed based on aligned sequences of DNA polymerases (Fig. 2). All sequences were collected from NCBI and aligned using CLUSTALW [24]; their evolutionary analysis was inferred through the neighbor-joining method using MEGA6 [25].

**Table 3:** Genome statistics

Attribute	Value	% of the Total^a^
Genome size (bp)	80,578	100.00
DNA coding (bp)	72,239	90.00
DNA G + C (bp)	32,674	40.55
DNA scaffolds	0	0.00
Total genes	103	100
Protein coding genes	93	90.29
RNA genes	10	9.71
Pseudo genes	0	0.00
Genes in internal clusters	0	0.00
Genes with function prediction	34	36.56
Genes assigned to COGs	33	35.49
Genes with Pfam domains	0	0.00
Genes with signal peptides	1	1.08
Genes with transmembrane helices	1	1.08
CRISPR repeats	1	-

^a^The total is based on either the size of the genome in base pairs or the total number of protein-coding genes in the annotated genome

**Table 4:** Number of genes associated with general COG functional categories

Code	Value	% of Total^a^	Description
J		0	Translation, ribosomal structure and biogenesis
A		0	RNA processing and modification
K	1	1,08	Transcription
L	13	13,98	Replication, recombination and repair
B		0	Chromatin structure and dynamics
D		0	Cell cycle control, Cell division, chromosome partitioning
V		0	Defense mechanisms
T		0	Signal transduction mechanisms
M		0	Cell wall/membrane biogenesis
N		0	Cell motility
U		0	Intracellular trafficking and secretion
O		0	Posttranslational modification, protein turnover, chaperones
C		0	Energy production and conversion
G		0	Carbohydrate transport and metabolism
E		0	Amino acid transport and metabolism
F	1	1,08	Nucleotide transport and metabolism
H		0	Coenzyme transport and metabolism
I		0	Lipid transport and metabolism
P		0	Inorganic ion transport and metabolism
Q		0	Secondary metabolites biosynthesis, transport and catabolism
R		0	General function prediction only
S	59	63,44	Function unknown
-	1	1,08	Not in COGs
X	18	19,35	Mobilome: Prophage, Transposons

^a^The total is based on the total number of protein coding genes in the genome

## Insights from the genome sequence

**Fig. 4:**
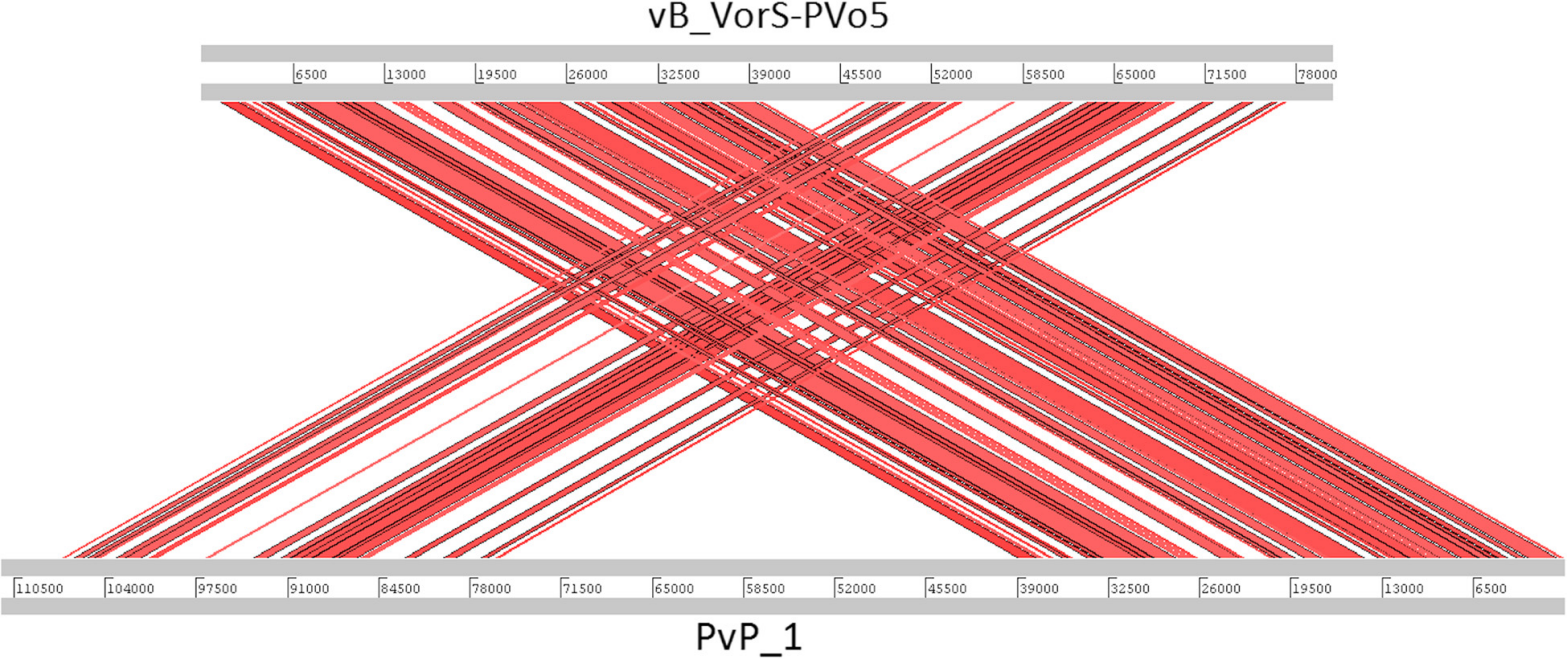
Whole genome comparison between *Vibrio* phage vB_VorS-PVo5 and *Vibrio* phage pVp_1. This figure was generated using the Artemis Comparison Tool (ACT)

Based on its morphological characteristics, the phage vB_VorS-PVo5 was attributed to the family of the Siphoviridae. Gene comparisons confirmed the relatedness to other members in the family. 93 ORFs code for proteins homologous to known phages, 82 of which show similarity with *Vibrio* phage pVp-1, (GenBank accession number JQ340389), indicating a close genetic relationship between these two phages (Fig. 4). The phylogenetic tree comparing DNA-Polymerase genes confirms this similarity, forming a robust cluster supported by high bootstrap values (Fig. 3), and therefore suggests a high degree of relatedness between vB_VorS-PVo5 and other *Vibrio* phages. A whole genome comparison of *Vibrio* phage vB_VorS-PVo5, and *Vibrio* phage pVp-1, was performed using the Artemis Comparison Tool [35]. Genomes were aligned by WebACT, using the default tblastx settings (E-value: 10e-4, genetic code: eubacterial). The ACT display was set to show homologous regions with BLAST scores of > 40, and a sequence similarity of >25 %.

Phage reproduction may involve either a lytic or a lysogenic cycle with some viruses being capable of performing both. In the lysogenic cycle the viral genome will integrate with host DNA and replicate along with it, whereas the lytic phage will destroy the host cell immediately after the replication, breaking bacterial cells open and allowing the phage progeny to find new hosts to infect. Due to quickly destroying the bacterial cells, lytic phages are more suitable for phage therapy, and the genes coding for the production of endolysin are the key evidence for the phage’s lytic characteristics [36, 37]. A BLASTP comparison of the endolysin sequences displayed a similarity of 94 % and 46 % with the *Vibrio* phage pVp-1 and *Vibrio* phage Phi-3, respectively. Furthermore, the enzyme destroys bacterial cell walls and has therefore been discussed for its use as an anti-infective to control pathogens [36, 38].

The specificity of the phage vB_VorS-PVo5 has been tested on *Vibrio anguillarum**,* the most closely related species to *Vibrio ordalii* [8] and another Chilean strain of *Vibrio ordalii**,* isolated from scallops, and whereas vB_VorS-PVo5 infected both *Vibrio ordalii* species it did not lyse *Vibrio anguillarum**.* More research has to be done in order to test the effect of vB_VorS-PVo5 phage and its endolysin as a therapy, however, the fact that only *Vibrio ordalii* strains were lysed indicates that vB_VorS-PVo5 might be highly species specific and may therefore prove to be a very promising candidate for phage therapy against *Vibrio ordalii*.

## Conclusions

Here we report for the first time on the isolation and genome sequencing of vB_VorS-PVo5 a novel phage that belongs to the family of the Siphoviridae and is capable of lysing the pathogen *Vibrio ordalii*ATCC 33509. The lytic character of the phage, together with the first indication of its specificity for *Vibrio ordalii* strains indicates the potential for its future use in aquaculture applications, controlling the pathogen either by using the phage or its endolysin.

## References

[1] Aravena G, Broitman B, Stenseth NC (2014). Twelve years of change in coastal upwelling along the Central-Northern Coast of Chile: spatially heterogeneous responses to climatic variability. PLoS ONE.

[2] Taylor MH, Wolff M (2007). Trophic modeling of Eastern Boundary Current Systems: a review and prospectus for solving the “Peruvian Puzzle”. Rev Peru Biol.

[3] Neale P, Sobrino C, Segovia M, Mercado J, Leon P, Cortés M, Tuite P, Picazo A, Salles S, Cabrerizo M, Prasil O, Montecino V, Reul A, and Fuentes-Lema A. Effect of CO2, nutrients and light on coastal plankton. I. Abiotic conditions and biological responses. Aquat Biol. 2014;22:25–41.

[4] Leyton Y, Riquelme C (2008). Vibrios en los sistemas marinos costeros. Rev Biol Mar Oceanogr.

[5] Paillard C, Le Roux F, Borrego JJ (2004). Bacterial disease in marine bivalves, a review of recent studies: trends and evolution. Aquat Living Resour.

[6] Bohle H, Kjetil F, Bustos P, Riofrío A, Peters C (2007). Fenotipo atípico de Vibrio ordalii, bacteria altamente patogénica aislada desde salmón del Atlántico cultivado en las costas marinas del sur de Chile. Arch Med Vet.

[7] Muller M, Ilardi P, Avendaño-Herrera R (2011). Eficacia de un desinfectante sobre Vibrio ordalii, Vibrio anguillarum, Francisella sp. y Virus de la necrosis Pancreática infecciosa (IPNV), patógenos de salmón del atlántico (Salmo salar) cultivado en Chile. Arch Med Vet.

[8] Schiewe M, Crosa J, T. Trust (1981). Vibrio ordalii sp. nov.: a causative agent of vibriosis in fish. Curr Microbiol.

[9] Silva-Rubio A, Acevedo C, Magariños B, Jaureguiberry B, Toranzo A, Avendaño-Herrera R (2008). Antigenic and molecular characterization of Vibrio ordalii strains isolated from Atlantic salmon Salmo salar in Chile. Dis Aquat Org.

[10] Alderman DJ, Hastings TS (1998). Antibiotic use in aquaculture: development of antibiotic resistance – potential for consumer health risks. Int J Food Sci Technol.

[11] Romero J, Feijoo CG, Navarrete P, Gianmarco Silva D, Silva RJ, Carvalho E (2012). Antibiotics in aquaculture – use, abuse and alternatives. Health and environment in aquaculture. Vol. 1.

[12] Cabello FC (2004). Antibióticos y acuicultura en Chile: consecuencias para la salud humana y animal. Rev Med Chil.

[13] Boyd CE, Massaut L (1999). Risks associated with the use of chemicals in pond aquaculture. Aquac Eng.

[14] Cabello FC (2006). Heavy use of prophylactic antibiotics in aquaculture: a growing problem for human and animal health and for the environment. Environ Microbiol.

[15] Palmer AC, Angelino E, Kishony R (2009). Chemical decay of an antibiotic inverts selection for resistance. Nat Chem Biol.

[16] Foster PL (2007). Stress-induced mutagenesis in bacteria. Crit Rev Biochem Mol Biol.

[17] Done HY, Halden RU (2015). Reconnaissance of 47 antibiotics and associated microbial risks in seafood sold in the United States. J Hazard Mater.

[18] Kutter EM, Kuhl SJ, Abedon ST (2015). Re-establishing a place for phage therapy in western medicine. Future Microbiol.

[19] Sarhan WA, Azzazy HM (2015). Phage approved in food, why not as a therapeutic?. Expert Rev Anti-Infect Ther.

[20] Verbeken G, Huys I, Pirnay J-P, Jennes S, Chanishvili N, Scheres J, Chanishvili N, Scheres J, Górski A, De Vos D, and Ceulemans C. Taking bacteriophage therapy seriously: a moral argument. BioMed Res Int. 2014;2014:8. 10.1155/2014/621316 PMC4020481 24868534

[21] Keen EC, Adhya SL (2015). Review of phage therapy: current research and applications. Clin Infect Dis.

[22] Chen F, Wang K, Huang S, Cai H, Zhao M, Jiao N, and Wommack KE. Diverse and dynamic populations of cyanobacterial podoviruses in the Chesapeake Bay unveiled through DNA polymerase gene sequences. Environ Microbiol. 2009;11(11):2884–92. 10.1111/j.1462-2920.2009.02033.x 19703219

[23] Kim JH, Jun JW, Choresca CH, Shin SP, Han JE, Park SC (2012). Complete genome sequence of a novel marine siphovirus, pVp-1, infecting vibrio parahaemolyticus. J Virol.

[24] Thompson JD, Higgins DG, Gibson TJ (1994). CLUSTAL W: improving the sensitivity of progressive multiple sequence alignment through sequence weighting, position-specific gap penalties and weight matrix choice. Nucleic Acids Res.

[25] Tamura K, Stecher G, Peterson D, Filipski A, Kumar S (2013). MEGA6: molecular evolutionary genetics analysis version 6.0.. Mol Biol Evol.

[26] Gratia A (1936). Numerical relations between lysogenic bacteria and particles of bacteriophage. Ann Inst Pasteur.

[27] Suttle CA, Chen F (1992). Mechanisms and rates of decay of marine viruses in seawater. Appl Environ Microbiol.

[28] Boyle JM, Symonds N (1969). Radiation-sensitive mutants of T4D I. T4y: A new radiation-sensitive mutant: Effect of the mutation on radiation survival, growth and recombination. Mutat Res Fundam Mol Mech Mutagen.

[29] Zhou Y, Liang Y, Lynch KH, Dennis JJ, Wishart DS. PHAST: A Fast Phage Search Tool. Nucleic Acids Res. 2011. 37. Effantin, G., P. Boulanger, E. Neumann, L. Letellier, and J.F. Conway, *Bacteriophage T5 structure reveals similarities with HK97 and T4 suggesting evolutionary relationships.* Journal of Molecular Biology, 2006. **361**(5): p. 993–1002. 10.1016/j.jmb.2006.06.081 16876823

[30] Delcher AL, Harmon D, Kasif S, White O, Salzberg SL (1999). Improved microbial gene identification with GLIMMER. Nucleic Acids Res.

[31] Schattner P, Brooks AN, Lowe TM (2005). The tRNAscan-SE, snoscan and snoGPS web servers for the detection of tRNAs and snoRNAs. Nucleic Acids Res.

[32] Krogh A, Larsson B, von Heijne G, Sonnhammer ELL (2001). Predicting transmembrane protein topology with a hidden Markov model: application to complete genomes. J Mol Biol.

[33] Petersen TN, Brunak S, von Heijne G, Nielsen H (2011). SignalP 4.0: discriminating signal peptides from transmembrane regions. Nat Methods.

[34] Lima-Mendez G, Toussaint A, Leplae R (2007). Analysis of the phage sequence space: the benefit of structured information. Virology.

[35] Carver TJ, Rutherford KM, Berriman M, Rajandream M-A, Barrell BG, Parkhill J (2005). ACT: the Artemis Comparison Tool. Bioinformatics (Oxford, England).

[36] Yang H, Yu J, Wei H (2014). Engineered bacteriophage lysins as novel anti-infectives. Front Microbiol.

[37] Wang I-N (2006). Lysis timing and bacteriophage fitness. Genetics.

[38] Fischetti VA (2010). Bacteriophage endolysins: a novel anti-infective to control Gram-positive pathogens. Int J Med Microbiol.

[39] King AM, Adams MJ, Carstens EB, Lefkowitz EJ (2012). Virus taxonomy: Ninth report of the International Committee on Taxonomy of Viruses.

[40] shburner M, Ball CA, Blake JA, Botstein D, Butler H, Cherry JM, Davis AP, Dolinski K, Dwight SS, Eppig JT, Harris MA, Hill DP, Issel-Tarver L, Kasarskis A, Lewis S, Matese JC, Richardson JE, Ringwald M, Rubin GM, and Sherlock G. Gene ontology: tool for the unification of biology. Nat Genet. 2000;25(1):25–9. 10.1038/75556 PMC3037419 10802651

